# Differential Diagnosis of Gastralgia and Other More Serious Affections of the Stomach

**Published:** 1851-03

**Authors:** 


*Differential Diagnosis of Gastralgia and other more Serious Affections of the Stomach.* By M. Valleix.—The diagnosis of gastralgia is often difficult; the slighter forms may be mistaken for the slight gastric disturbance, called by the French, "*embarras gastrique*," or for acute gastritis; while the ordinary chronic forms may be mistaken for chronic gastritis, simple ulcer of the stomach, cancer of the stomach, or intercostal neuralgia.

The most important distinctive signs are thus given by Valiex:

| GASTRALGIA—ACUTE FORM. | GASTRIC DISTURBANCE. |
|---|---|
| *Acute pain* in the epigastrium. | *Discomfort* rather than pain. |
| Appetite *preserved.* | *Anorexia.* |
| *No headache.* | *Headache* frequent. |
| Nausea *only after meals, or in the morning.* | Nausea frequent at *all periods of the day.* |

| GASTRALGIA—ACUTE FORM. | ACUTE GASTRITIS. |
|---|---|
| Appetite *good.* | Appetite *lost.* |
| *No pain* on pressure. | *Acute pain* on pressure. |
| Vomiting *rare, mucus* or of *food.* | *Bilious* vomiting *frequent.* |
| *No fever.* | *Distant fever.* |

| CHRONIC GASTRALGIA. | CHRONIC GASTRITIS. |
|---|---|
| *Usually uncomplicated.* | *Usually complicated* with another affection. |
| Vomiting *rare, mucus or food.* | *Bilious* vomiting frequent. |
| Spontaneous pains often *very acute.* | Spontaneous pains *less acute.* |
| *Usually no* pain on pressure. | *Pain* on pressure *acute.* |
| Progress of disease *irregular.* | Progress less irregular. |
| *Absence of fever.* | *Fever generally present.* |

| CHRONIC GASTRALGIA. | SIMPLE ULCER OF THE STOMACH. |
|---|---|
| Appetite *more or less preserved.* | Appetite *lost.* |
| Vomiting a *considerable period* after food. | Vomiting *immediately* after food. |
| *No* vomiting of pure blood or dark matter. | *Sometimes* vomiting of blood or black matter. |
| Progress *slow.* | Progress *rapid.* |

| CHRONIC GASTRALGIA. | CANCER OF THE STOMACH. |
|---|---|
| Vomiting as before. | Vomiting *at long periods after food.* |
| Destroys *slowly.* | Destroys *rapidly.* |
| *No signs* of cancerous cachexy. | *Signs* of cancerous cachexy. |
| Progress *irregular.* | Progress *regular.* |

| CHRONIC GASTRALGIA. | INTERCOSTAL NEURALGIA. |
|---|---|
| Pain *not increased by pressure.* | Pains *on pressure.* |
| Well marked gastric disturbance. | No gastric disturbance. |

*British and Foreign Medico-Chir. Review.—Ib.*

